# The Brain Worker and His Food

**Published:** 1917-03-17

**Authors:** 


### THE BRAIN WORKER AND HIS FOOD.

With the general principles of wise feeding we dealt in an article published in our issue of March 3, p. 433, and, having prominently before us the case of the industrial worker, we insisted on the need for an adequate supply of the essential foods if a high level of efficiency is to be maintained. What is needed for mere existence is one thing, but to add achievement to life makes a new demand. Besides the industrial population there is another class on whom the pressure of modern circumstances is inflicting an added strain. We refer to the brain worker who has accepted a larger burden than he has been accustomed to carry, perhaps because events give him little choice in the matter, perhaps from the patriotic desire to set free for active tasks those whose years are appropriate to these duties. There is no doubt that under the conditions created by the war many elderly men have resumed responsibilities from which they had reasonably freed themselves, and are at present, both as regards the nature and duration of their work, systematically discharging duties which mean a serious strain on their nerve force. Accustomed to intellectual exercise free from pressure, they find themselves again engulfed in the world of affairs and driven at a speed to which they had become unaccustomed. Little wonder if to men so situated the food question—the voice of the official Controller apart—sometimes takes on an anxious aspect. A consciousness of the strain under which they are living is a daily experience, and they ask themselves whether the food habits of easier times are adequate to the newer demands.

Regarding the position generally we should say that the workers with whom we are here concerned ought, in the first place, to be warned against the generous diet which seemed suitable to them in their earlier working days. They have gone back to junior habits as workers, but they must not follow a similar line in reference to food. Time has had its influence, and the assimilative and excretory capacities of the body have declined. To attempt therefore to meet the new conditions by reverting to the more stimulating nitrogenous foods, such as red meats and animal soups, is worse than useless. It is, indeed, to steer towards disaster. Under such influences there will almost certainly be a collapse in one direction or another. With white flesh as fish or fowl there may be a little more liberty, but even here great discretion ought to be exercised. It is a common delusion to place a deep line of separation between the two groups here contrasted and to label the one as wholly dangerous and the other as completely safe. The difference, such as it is, is one of degree not of kind, and though one class may be preferred to the other, neither admits of an unrestrained liberty. The brain worker will be unwise to try to meet the new strain in his life by liberal supplies of flesh foods, even though he elects for the less stimulating variety. Milk, eggs, vegetables, fruit, and the various carbo-hydrates are at his choice, and it is mainly from these that he must get the energy which he needs. Happy is he if a thoughtful interest and competent hands are ready to supply the skilful preparation which gives to food an added benediction.

But if there is a danger of excess there is also a danger of defect. Strictly speaking, additional work demands additional food, and instances occur where the practice of the simple life is found to be unequal to the new strain. Especially should this be prominent before the minds of those who have been accustomed to take only scanty meals and at long intervals. Under new demands such as are here contemplated, some modification of this practice is advisable, and small meals at more frequent hours should be accepted. There can be no hard-and-fast rule on such a point. But the man who has gone back to the tasks of former years will generally need some readjustment of his food habits, and this ought to take place on the lines which are here defined. If at the same time he keeps his excretory organs active by free draughts of diluent fluids, takes some daily walking exercise, and avoids the midnight oil, he will at least have done his best to deserve success, and we cordially hope he will achieve it.

